# An Efficient Approach for Inverting the Soil Salinity in Keriya Oasis, Northwestern China, Based on the Optical-Radar Feature-Space Model

**DOI:** 10.3390/s22197226

**Published:** 2022-09-23

**Authors:** Nuerbiye Muhetaer, Ilyas Nurmemet, Adilai Abulaiti, Sentian Xiao, Jing Zhao

**Affiliations:** Xinjiang Key Laboratory of Oasis Ecology, College of Geographical and Remote Sensing Sciences, Xinjiang University, Urumqi 830046, China

**Keywords:** soil salinity, Landsat 8 OLI data, PALSAR-2 data, Keriya Oasis, polarization decomposition

## Abstract

Soil salinity has been a major factor affecting agricultural production in the Keriya Oasis. It has a destructive effect on soil fertility and could destroy the soil structure of local land. Therefore, the timely monitoring of salt-affected areas is crucial to prevent land degradation and sustainable soil management. In this study, a typical salinized area in the Keriya Oasis was selected as a study area. Using Landsat 8 OLI optical data and ALOS PALSAR-2 SAR data, the optical remote sensing indexes NDVI, SAVI, NDSI, SI, were combined with the optimal radar polarized target decomposition feature component (VanZyl_vol_g) on the basis of feature space theory in order to construct an optical-radar two-dimensional feature space. The optical-radar salinity detection index (ORSDI) model was constructed to inverse the distribution of soil salinity in Keriya Oasis. The prediction ability of the ORSDI model was validated by a test on 40 measured salinity values. The test results show that the ORSDI model is highly correlated with soil surface salinity. The index ORSDI_3_ (R^2^ = 0.656) shows the highest correlation, and it is followed by indexes ORSDI_1_ (R^2^ = 0.642), ORSDI_4_ (R^2^ = 0.628), and ORSDI_2_ (R^2^ = 0.631). The results demonstrated the potential of the ORSDI model in the inversion of soil salinization in arid and semi-arid areas.

## 1. Introduction

Soil salinization is a severe global environmental disaster, especially in arid and semi-arid areas [1,2,3]. Soil salinity is a result of the action of the combination of many natural factors, including harsh climate, topography, hydrogeology, and anthropogenic factors such as agricultural irrigation [4,5,6,7]. Soil salinization remarkably triggers soil erosion, declining agricultural productivity, and restricting the stability of the ecological environment [8,9]. Recent estimates have shown that salinity affects 20% of the world’s irrigated land, and this proportion is an increasing trend [1]. It has been estimated that by 2050, the salinity of global arable cultivated land will reach more than 50% [10,11,12]. With the intensification of global warming, the problem of soil salinization has become more prominent in low and middle latitudes regions [13,14]. China has more than 30% of the world’s saline soil [15], with a total saline soil area of approximately 3600 × 10^4^ hm^2^, accounting for 4.88% of the country’s available land area [16]. According to a survey by the Xinjiang Institute of Ecology and Geography of the Chinese Academy of Sciences conducted in 2014, the arable land affected by salinity in Xinjiang accounted for approximately 37.72% of the country’s total irrigated area [17]. Xinjiang province includes 60.6% of China’s total saline-alkali land area, and soil salinization is more prominent in the southern part of the province [18]. One typical saline-soil area is Keriya River Basin, a coupled oasis–desert ecosystem in southern Xinjiang. Salinization has been the most severe land degradation process in the Keriya oasis, leading to a gradual decline in its crop production. Hence, the timely and effective monitoring of soil salinization in this region is of great significance to the improvement and management of soil salinity.

Soil salinization is a complex dynamic process, which makes its detection, dynamic monitoring, and mapping difficult [6]. The traditional methods for soil salinity analysis include field soil sample investigation and analysis [19], which are laborious and require many materials, and financial resources [20,21]. These methods are also limited to a small area, unable to realize large-scale real-time dynamic monitoring [19], large-scale salinization monitoring and evaluation lack timeliness and representativeness [22], so only a small amount of observational information can be obtained [20,22]. Remote sensing technology can simultaneously observe information from the same area over various scales and has the characteristics of wide coverage [19], high spatial resolution, integration, dynamics, and short revisit periods [19,20,23,24]. Soil information can be extracted rapidly and accurately [25]. With the continuous development of remote sensing technology, it has become possible to obtain information on salinization in the Keriya Oasis.

Currently, various satellite remote sensing data with medium and high spatial and temporal resolutions have been widely used in identifying and monitoring salt-affected areas and monitoring various properties and changes in soil characteristics [1,26]. Several studies have shown that the visible, near-infrared, and other bands of optical sensors have promising application prospects in monitoring and identifying surface soil salinity [27,28,29,30,31]. The Landsat 8 OLI data are suitable for monitoring salt-affected soils and represent an important data source for soil salinity monitoring [32,33]. Based on the spectral characteristics of vegetation and soil salinity, many studies have proposed various spectral characteristic indexes (e.g., vegetation and soil salt indexes) that highlight surface characteristics [22]. Khan et al. [34]. defined three salinity indexes suitable for inversion of salinization in arid areas: the salinization index (SI-T), brightness index (BI), and normalized difference salinity index (NDSI) [28]. The presence of saline plants in saline areas and adaptive changes in plant morphology under salt stress make the vegetation index an indirect indicator of the salinity degree. The normalized difference vegetation index (NDVI) has been widely used in the inversion of soil salinity [35,36]. Both the vegetation and salinity index have achieved satisfactory results in salinity monitoring [22].

Usually, salt is concentrated in the soil profile underlying the surface of vegetation cover, so it can be difficult to detect using optical remote sensing [37,38]. Synthetic aperture radar (SAR)-based imaging can be used for reliable salinity monitoring due to its sensitivity to soil electrical conductivity (EC) [39,40,41,42], its independence from atmospheric conditions, and its ability to penetrate the subsoil to a depth of more than 50–150 cm [43]. Microwave remote sensing represents an effective method for evaluating soil salinization [44] and has a great potential for assessing soil salinity [45,46]. However, few attempts have been made to use radar to detect soil salinity [43,47,48,49].

Unlike single and dual-polarization, fully-polarimetric SAR data contain a target’s scattering matrix, dielectric constant information, and more geometric details of the target, and are also sensitive to the geometry and height of the surface vegetation scatterers, making it able to compensate for the shortcomings of optical remote sensing [50]. Therefore, fully-polarimetric SAR data may be applied to the classification and mapping of soil salinity [51].

In recent years, many studies have monitored soil salinization and soil desertification by using the feature space model of the interaction between two optical surface parameters. J. Liu et al. [52] proposed Albedo-MSAVI, SI-Albedo and SI-NDVI feature space models and found that the above models could better invert the salinization status of typical saline soils in China. Guo et al. [53] based on optical remote sensing images, obtained five typical desertification indexes and constructed 10 feature space models to build desertification monitoring indicators and found that the research results can provide a reference for desertification control decisions. However, most studies are based on a single data source, i.e., optical remote sensing images, and fewer studies have used multi-source remote sensing data to construct feature spaces.

In this study, the main objective was: (1) to introduce fully-polarimetric radar data to construct feature space models of different degrees of salinity (mild, moderate and severe); (2) to explore the potential of optical surface parameters and radar polarization feature components for synergistic inversion of soil salinity; (3) to produce soil salinity maps with multi-source remote sensing data in typical salinized areas.

## 2. Study Site and Data

### 2.1. Study Site Description

**Figure 1:**
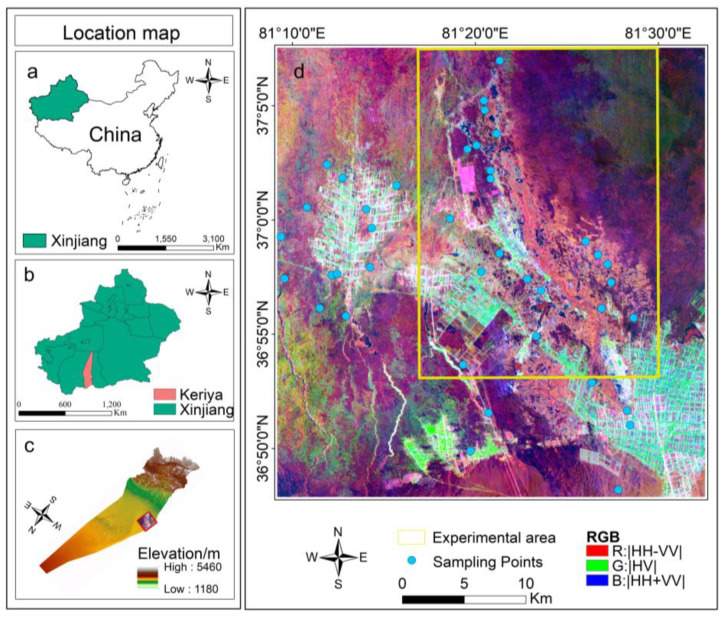
Map of the study area. (**a**) Northwestern China; (**b**) Keriya Oasis in Xinjiang; (**c**) study area in Keriya Oasis; (**d**) PALSAR-2 image of study area.

The Keriya Oasis in Xinjiang Province is adjacent to the Taklamakan Desert in the south and Kunlun Mountain in the north and distributed along midstream and downstream of the Keriya River Basin (36°47′–37°7′ N, 81°09′–81°45′ E) [54,55], as shown in Figure 1. The Keriya oasis has a total area of 4.03 × 10^4^ km^2^ [56], east-west width of 30 km–120 km, and a north-south length of 466 km. With an average annual precipitation of only approximately 14 mm and evaporation of up to 2500 mm, the Keriya oasis is mainly irrigated by snow and ice melt, as well as groundwater in the mountains [57]. The landscape is dominated by denuded pre-mountain sloping gravelly plains, alluvial plains, and piled desert landscapes. Due to the deep inland location and mountain basins, this region has a warm temperate continental arid desert climate, with sufficient sunlight and abundant heat and an average annual temperature of 12.4 °C [54]. The main land use in the study area is agriculture, and the main crop is cotton; the natural vegetation is mainly reeds, tamarisk willow, poplar, camel thorn. The main land cover types include rivers, farmland, swamps, reservoir, deserts, Gobi, and shrubs. The soil type is mainly meadow soil and brown desert soil, with poor permeability of soil granules and different degrees of soil salinity [51].

A seasonal river, the Keriya, flows through the Keriya Oasis and disappears in the hinterland of the Taklamakan Desert [58]. Due to the long-term mutual influence of the arid continental climate and the mountain-basin landscape pattern, the Keriya oasis has formed a mature oasis-desert ecosystem, which occupies an extremely important position in the study of the environmental evolution of arid zones [59].

### 2.2. Data

#### 2.2.1. Satellite Data Acquisition and Pre-Processing

The Landsat 8 OLI data have a revisit period of 16 days, which provides a great advantage for monitoring saline soil [22]. The optical satellite data used in this study were obtained from Landsat 8 OLI data (28 May 2015) collected in the study area (available at http:/www.gscloud.cn/, accessed on 29 March 2022) with a cloud cover of less than 10%. Furthermore, ALOS PALSAR-2 single-look complex (SLC) images were acquired to investigate the potential of using polarized SAR data to monitor saline soils in extremely arid regions. The ALOS PALSAR-2 SAR data used in this study were collected in the descending orbit on 23 April 2015, when the satellite was operating in quadrupole stripe mode, including HH, HV, VH, and VV polarization, and passed through the sampling area at an incident angle of 30.4°. The ALOS PALSAR-2 satellite is equipped with a phased array L-band synthetic aperture radar sensor, which can work around the clock under any atmospheric conditions, and thus has been widely applied to the fields of regional observation and disaster monitoring [60,61].

The Landsat 8 OLI data of the study area were preprocessed using ENVI 5.6^®^ software. The preprocessing steps included: (1) radiometric correction, which converts an image’s Digital Number (DN) values to radiance values; (2) atmospheric correction, which eliminates the effects of factors such as atmosphere and light on the reflection of ground objects; (3) image resizing, which is used to achieve an optimal image resolution of 15m × 15 m; and (4) spatial subset, which subset out the study area and typical land type experimental area.

The ALOS PALSAR-2 data were preprocessed using the SNAP 8.0 software. The preprocessing steps included: (1) radiometric calibration; (2) multi-looking; (3) speckle filtering; (4) geocoding; (5) image resizing to the optimal resolution of 15 m × 15 m; (6) spatial subset, which subset out the study area and typical land type experimental area.

#### 2.2.2. Field Sampling and Laboratory Measurements

To ensure that the field sampling occurred when the satellite passed through the study area, this study conducted a 10-day field data sampling from 20 April 2015, to 1 May 2015. Soil sampling areas in the study area were selected by visual analysis of the Landsat 8 OLI images and based on fieldwork. Sampling points were randomly selected in each sampling area. Field sampling was conducted under dry weather conditions, and no rainfall was reported in the study area. Surface and subsurface (0–100 cm) soil samples were collected at each sampling site, and the corresponding GPS coordinates were recorded. The soil samples were stored in their natural state in sealed aluminum boxes to be taken to the laboratory for salinity testing.

Forty soil samples were analyzed in the laboratory, they were naturally dried, ground, and filtered through a 1-mm pore size sieve. Then, 50 g soil samples were weighed with an electronic balance, placed in a flask, and 250 mL of deionized water was added to configure a 5:1 water-to-soil ratio leaching solution. The solution was shaken by hand for approximately 3 min to mix it thoroughly. The completely mixed solution was left to stand for about 30 min, and when the solution become clear, it was filtered and extracted for the later determination of soil physicochemical properties. Soil salinity was determined using an Orion 115A+ instrument. To eliminate representative sampling errors, the mean of the measured values of three samples from each sampling site was used as a representative value for that site.

The obtained salinity results were tabulated and compared with the “Work Outline about Reuse of Salted Soil at the County Level in Xinjiang” provided by the Xinjiang Water Resources Department, as shown in Table 1.

## 3. Methodology

**Figure 2:**
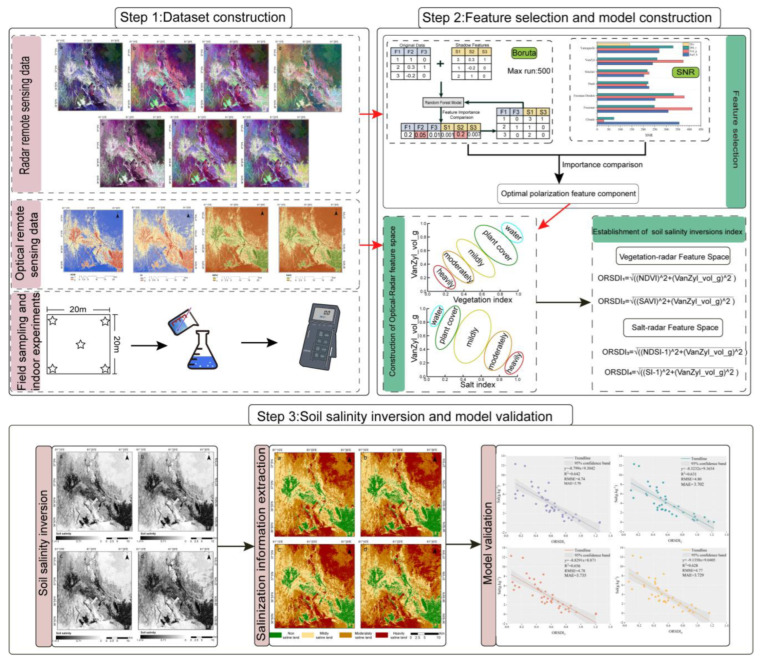
The overall workflow of the study.

In this paper, four typical surface parameters (NDVI, SAVI, NDSI, and SI) based on LANDSAT-8 OLI image were calculated by using the Band math module of ENVI 5.6. In addition, by using SNAP 8.0 software, seven polarization decompositions based on ALOS PALSAR-2 image were calculated and 22 polarization feature components were obtained. The optimal polarized feature component was obtained using the Boruta feature selection algorithm as well as signal noise ratio (SNR). The optical-radar based salinity inversion model was then constructed with the support of two-dimensional feature space theory, which was utilized to obtain the spatial distribution map. The corresponding inversion accuracies and their comparisons were also carried out based on 40 measured samples. The main steps followed in this study are shown in Figure 2.

### 3.1. Polarization SAR Target Decomposition

From the polarized SAR image data, the polarized scattering characteristics of the target can be extracted so as to realize other operations such as classification, detection, and the recognition of fully-polarized data. This requires effectively analyzing the polarization data and extracting the scattering characteristics of the target, which is based on the target polarization decomposition.

Huynen [62] first proposed the theory of target polarization decomposition. The basic principle of the polarization target decomposition is to decompose the scattering matrix, covariance matrix, or Mueller matrix into a number of physically meaningful sums of scattering mechanisms [63,64]. The decomposition is helpful to effectively extract the scattering features of the target. Polarization target decomposition methods can be roughly classified into two categories: coherent target decomposition (CTD) and incoherent target decomposition (ICTD) [63].

The CDT represents a decomposition of the target scattering matrix, which requires the scattering characteristics of the target to be deterministic or steady-state and the scattered echoes to be coherent. The CTD methods include Pauli decomposition [65], Krogager decomposition [66], Cameron decomposition [67], and Sinclair decomposition [68], etc. The ICTD represents a decomposition of the polarized covariance matrix, polarized coherence matrix, Muller matrix, or Stokes matrix; the target scattering is non-deterministic (or time-varying) and incoherent (or partially coherent) at the time of echo. The ICTD methods include Huynen decomposition [62], Cloude decomposition [69], Holm and Barnes decomposition [70], and Freeman Durden decomposition [71], etc.

A variety of polarized scattering components can be obtained using the target decomposition method, such as surface, double bounce, volume, and helix scattering. In the polarization decomposition, the double scattering component and the surface scattering component represent the backward scattering information of tree trunk and ground surface obtained from the SAR signal through the canopy; vegetation area dominated by volume scattering [71]; the helix component is stronger in areas with vegetation, but remains relatively small overall, rarely exceeding 10% scattering [72]. For vegetated areas, the main scattering mechanisms are generally assumed to be direct scattering from branches with randomly-distributed directions, two reflections from the combination of the ground and tree trunks, and surface reflection from the ground (it should be noted that this latter reflection is weaker than the others) [72].

To make full use of the PolSAR data, several polarization decomposition methods have been proposed, and the corresponding polarization information has been extracted. The decomposition methods that were investigated in this research include the methods of Cloude, Freeman, Freeman Durden, Pauli, Sinclair, VanZyl, and Yamaguchi. The physical explanation and detailed calculation process of these polarization parameters can be found in [73].

**Figure 3:**
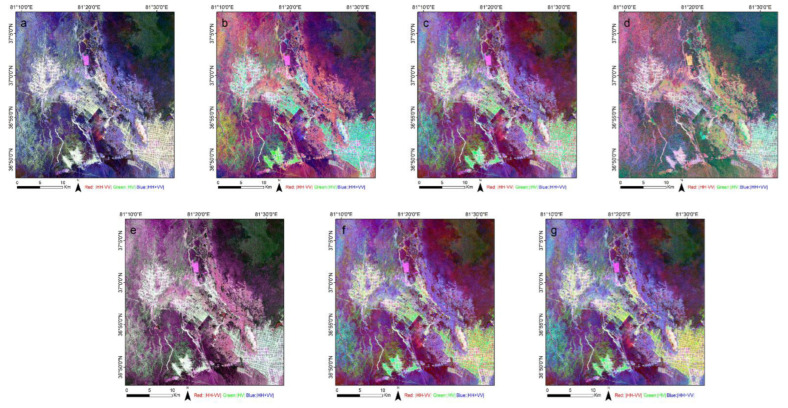
Polarization decomposition results of the RGB standard composite images obtained by different methods: (**a**) Pauli; (**b**) Freeman; (**c**) Freeman Durden; (**d**) Cloude; (**e**) Sinclair; (**f**) VanZyl; (**g**) Yamaguchi.

Finally, a total of 22 polarization features were obtained from the ALOS PALSAR-2 images, as e given in Table 2. The standard RGB (R:|HH-VV|, G:|HV|, B:|HH+VV|) composite images showing some of the polarization decompositions are presented in Figure 3.

### 3.2. Typical Surface Parameters of Saline Soil Spectral Response

Reasonable selection of typical surface parameters of the spectral response of salinized soil is crucial to extracting the thematic information on salinization. Remote sensing sensors use electromagnetic waves reflected from the ground targets to collect information on saline soils [74]. The spectral indexes established using different combinations of bands can establish correlations with specific targets [75]. Since saline soil with different degrees of salinity show different spectral characteristics in the blue, green, red, and near-infrared bands in remote sensing images, a large number of salinity indexes have been proposed for soil monitoring and the mapping of salinity [75].

**Figure 4:**
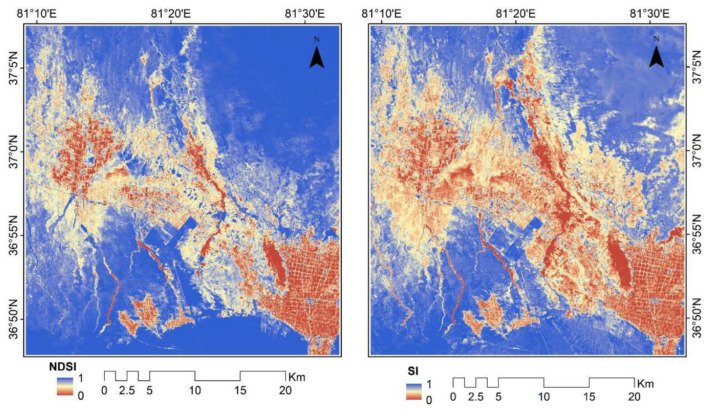
Salt index extraction results.

The salinity indexes used in this study include the salinity index (SI) and normalized differential salinity index (NDSI). Khan [34] found that the soil salinization degree can be accurately reflected by the SI through the analysis of band mixing experiments and comparison of spectral characteristics of typical features. The salinity index extraction results are shown in Figure 4.

**Figure 5:**
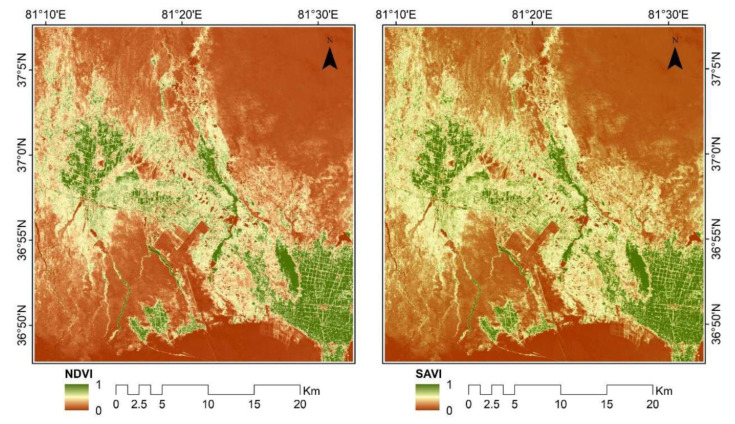
Vegetation index extraction results.

Since soil salinity affects vegetation, vegetation can be used as an indirect indicator of soil salinity [23,76]. Therefore, several studies have used various vegetation indexes to assess soil salinity based on the vegetation reflectance [25]. The vegetation indexes used in this paper include the normalized difference salinity index (NDVI) and the soil-adjusted vegetation index (SAVI). The NDVI is an effective and rapid indicator for identifying vegetation areas and can be used as an indicator to characterize the state of the environment [77]. The reason for choosing the SAVI as one of the vegetation indexes is that the SAVI can explain the changes in the optical characteristics of the soil background and correct the sensitivity of the soil background to the spectral index, thus minimizing the spectral variations caused by the soil background [78]. The results of vegetation index extraction are shown in Figure 5.

For the above-mentioned reasons, the SI, NDSI, NDVI, and SAVI were selected as important typical surface parameters to reflect the soil salinization degree. The salinity and vegetation indexes used in this study are shown in Table 3.

### 3.3. Optimal Polarization Component

The polarization features of PolSAR images include the inherent scattering mechanism of terrain type, which is important for terrain classification and other Earth-related observation applications [80]. However, the extracted parameters can include redundancy information and scattering noise, so not all the extracted feature parameters are suitable for the inversion of soil salinity [51]. Meanwhile, using the target decomposition method, a variety of polarization features can be extracted [51,80], if all of them are used as input data in the feature inversion process, the computational cost can significantly increase. Although all these features obtained from the coherence or covariance matrix are not independent [80], selecting features important for the inversion of different soil types can improve the accuracy of the soil salinity inversion.

To minimize the effects of human subjective factors, this study uses the Boruta feature selection algorithm, maximum signal-noise-ratio (SNR) methods to select a feature parameter that has less image noise and importance for the measured salinity (Sal) for monitoring and visualization of soil salinity.

#### 3.3.1. Polarization Feature Component Selection Algorithm

Not all polarized feature components are favorable for soil salinity inversion, so their importance needs to be assessed. In this study, we applied the Boruta algorithm, which is a wrapper algorithm based on random forests [81]. The idea of Boruta’s algorithm is that the original features are shuffled to construct shadow features, the original and shadow features are stitched together into a feature matrix for training, and finally, the feature importance of the shadow features is used as a reference base to select the set of features that are truly relevant to the dependent variable [82], the workflow of Boruta’s feature selection algorithm is shown in Figure 2.

**Figure 6:**
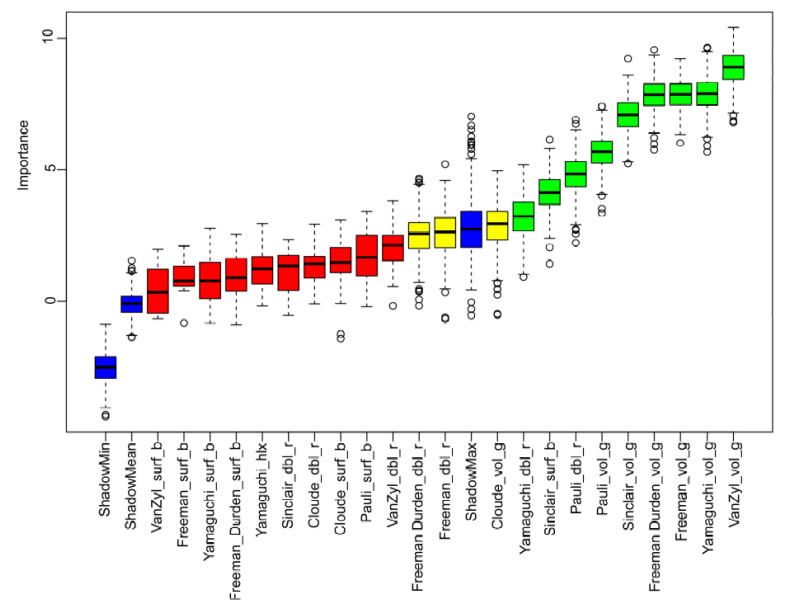
Importance of polarized feature components calculated by the Boruta algorithm. The blue boxplots are shadow features. Green, yellow, and red boxplots represent important, tentative, and unimportant variables, respectively.

All 22 polarization feature components obtained from 7 polarization decompositions were input into the Boruta model, in order to avoid the error caused by randomness, 500 iterations of feature importance calculation were carried out, after 500 iterations, a total of 22 polarization feature components were identified, whereas 9 polarization feature components were regarded as important for Sal (Figure 6). In general, the volume component extracted by polarization decomposition has a stronger relationship with Sal than the surface and double components. VanZyl_vol_g had the strongest relationship with the Sal, Yamaguchi_vol_g ranked next.

#### 3.3.2. SNR of Polarized Feature Components

For radar images, the removal of image noise is the key to accurate acquisition of object information, and the signal noise ratio can determine whether the image quality has been improved after denoising the image containing the image. Therefore, in this study, SNR is introduced as one of the evaluation indicators for selecting the optimal feature component. The SNR were calculated with IDL 8.7.3^@^ software.

The SNR of each polarization decomposition feature component was calculated, and then the SNR values of the three components of the same polarization decomposition were compared. Since the higher the image SNR, the better the image quality and de-noising effect [83], the components with the largest SNR were selected. The feature component with the highest SNR was selected from the three feature components that were extracted by each of the polarization decomposition methods, and seven feature components obtained by the seven polarization decomposition methods were selected, including Pauli_surf_b, Freeman_vol_g, Freeman Durden_vol_g, Cloude_surf_b, Sinclair_vol_g, VanZyl_vol_g, and Yamaguchi_dbl_r. Detailed information is shown in Table 4.

**Figure 7:**
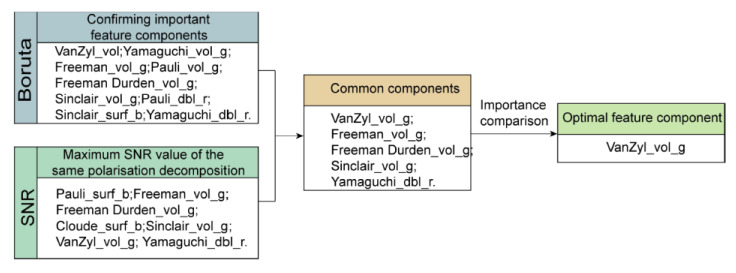
The optimal feature component selection process.

The importance of the common polarization feature components selected by the Boruta feature algorithm and SNR is compared, and the most important polarization feature components VanZyl_vol_g are selected as the optimal feature components, The optimal feature component selection process is shown in Figure 7.

### 3.4. Data Normalization

To eliminate the effect of variability in the magnitude order and unit between data on different variables, the data were standardized for the NDVI, SAVI, SI, NDSI, and VanZyl_vol_g. First, the minimum and maximum values of the NDVI, SAVI, SI, NDSI, and Van_vol_g in the study area were determined, and then these data were used for data normalization as follows:

NDVI = [(NDVI − NDVI_min_)/(NDVI_max_ − NDVI_min_)] (1)

SAVI= [(SAVI − SAVI_min_)/(SAVI_max_ − SAVI_min_)] (2)

SI = [(SI− SI_min_)/(SI_max_− SI_min_)] (3)

NDSI = [(NDSI− NDSI_min_)/(NDSI_max_− NDSI_min_)] (4)

Van_vol_g = [(Van_vol_g − Van_vol_g_min_)/(Van_vol_g_max_ − Van_vol_g_min_)] (5)

### 3.5. Principle of Feature Space

The feature space is a spatial system consisting of two or more typical surface parameters derived from satellites images [53,84]. In recent years, two-dimensional feature space models constructed through synergistic relationships between typical surface parameters have been found to have the potential for monitoring soil moisture, drought, and soil salinity [85,86]. They provide a good reference for soil salinity monitoring. However, most feature space models only use single remote sensing data such as optical images, ignoring other important remote sensing data such as radar images, which have great potential in the inversion of soil salinity. At present, there are few studies on the comprehensive use of optical remote sensing images and radar images to construct a feature space model of arid areas to inverse soil salinity. In this paper, a two-dimensional feature space is constructed using optical typical surface parameters as horizontal coordinates and radar polarization feature components as vertical coordinates in order to explore the potential of optical and radar data to synergistically invert soil salinity.

## 4. Construction of Different Feature Spaces and Inversion Models

### 4.1. Feature Space Construction

To analyze the distribution characteristics of soil salinity in the study area using the field survey data and OMap (available at https://www.ovital.com, accessed on 15 March 2022) as a reference, an experimental area with a typical land type was selected, as shown in Figure 1d. In this area, the soil salinity was high, and the interlacing zones of the mildly saline land to the moderately and heavily saline lands were evident.

Based on the aforementioned four typical surface parameters and one radar feature component, four optical-radar feature spaces were constructed. The four feature spaces were divided into two categories—the vegetation-radar feature space and the salt-radar feature space—depending on the optical typical surface parameters.

#### 4.1.1. Vegetation-Radar Feature Space

**Figure 8:**
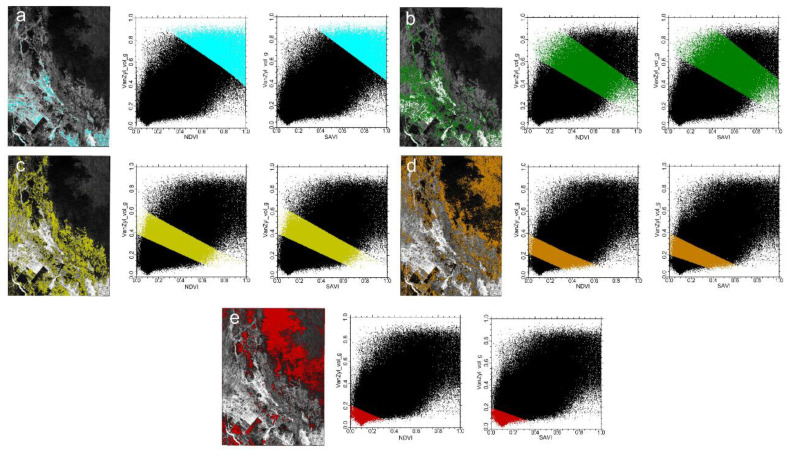
The soil salinity content in the images and NDVI-Van_vol_g and SAVI-Van_vol_g spaces: (**a**) water; (**b**) plant cover; (**c**) mildly saline soil; (**d**) moderately saline soil; (**e**) heavily saline soil.

The NDVI-Van_vol_g and SAVI-Van_vol_g feature spaces were constructed by utilizing the NDVI and SAVI indexes and Van_vol_g radar feature parameters. As shown in Figure 8, in the feature space, the vegetation index and the radar feature component (VanZyl_vol_g) had a significant positive correlation with each other, and there were obvious regularities for the different salinized soils in the formed feature space. The scatter points with different soil salinity degrees were concentrated in different parts of the scatter plot; as the vegetation cover increased, the value of the Van_vol_g component also increased, and the soil salinity degree tended to decrease. In Figure 8, blue areas denote water bodies; green areas mainly indicate agricultural cultivation areas; yellow areas mainly represent lightly saline soil; brown areas indicate moderately saline soil; and red areas denote heavily saline soil.

#### 4.1.2. Salt-Radar Feature Space

**Figure 9:**
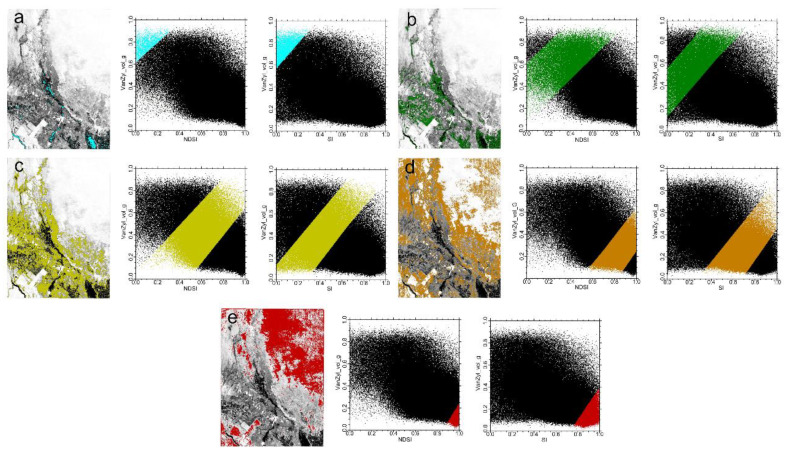
The soil salinity content in the image and NDSI-Van_vol_g and SI-Van_vol_g spaces: (**a**) water; (**b**) plant cover; (**c**) mildly saline soil; (**d**) moderately saline soil; (**e**) heavily saline soil.

By using the SI and NDSI indexes and Van_vol_g radar feature parameters, the NDSI-Van_vol_g, and SI-Van_vol_g feature spaces were constructed. As shown in Figure 9, the salt index and the radar feature component (VanZyl_vol_g) in the feature space had a significant negative correlation, and in the formed feature spaces, different salinized soils showed significant regularities; soils with different salinity degrees were concentrated in different sections of the typical study area. When the value of VanZyl_vol_g increased, the salinity index and the soil salinity decreased, and vice versa. In Figure 9, blue areas are water bodies; green areas mainly denote the plant cover; the yellow areas mainly represent lightly saline soil; brown areas indicate moderately saline soil; red areas denote heavily saline soil.

### 4.2. Inversion Models

**Figure 10:**
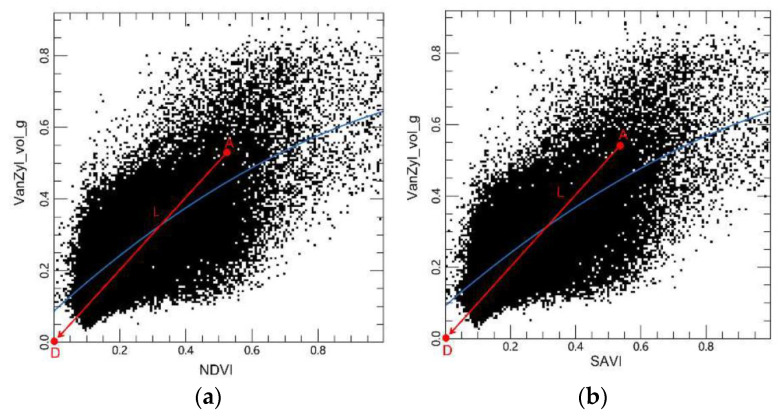
Construction of the vegetation-radar models: (**a**) NDVI-VanZyl_vol_g feature space; (**b**) SAVI-VanZyl_vol_g feature space. (The blue line is the trend line between the components; the red line L is the distance from any point A in the feature space to D (0,0)).

A simplified diagram of the significant spatial differentiation in the vegetation-radar feature space for different salinity degrees (i.e., water bodies, plant cover, mild salinity, moderate salinity, and heavy salinity) is presented in Figure 10. With the increase in the NDVI and SAVI indexes and VanZyl_vol_g component, the soil salinity decreased. The closer the distance from a point-to-point D (0, 0) in the feature space, the sparser was the plant cover and the higher the soil salinity. Based on the linear relationship, a remote sensing inversion model for soil salinity was established. The distance *L* from point A, which can be any point in the NDVI-Van_vol_g and SAVI-Van_vol_g feature spaces, to point D can be expressed by:

(6) $$L=\sqrt{{\left(\mathrm{NDVI}\right)}^{2}+{\left(\mathrm{VanZyl}\_\mathrm{vol}\_g\right)}^{2}}$$

(7) $$L=\sqrt{{\left(\mathrm{SAVI}\right)}^{2}+{\left(\mathrm{VanZyl}\_\mathrm{vol}\_g\right)}^{2}}$$

The Optical-Radar Salinity Detection Index (ORSDI) based on the NDVI-Van_vol_g and SAVI-Van_vol_g feature space can be, respectively, constructed as follows:

(8) $${\mathrm{ORSDI}}_{1}=\sqrt{{\left(\mathrm{NDVI}\right)}^{2}+{\left(\mathrm{VanZyl}\_\mathrm{vol}\_g\right)}^{2}}$$

(9) $${\mathrm{ORSDI}}_{2}=\sqrt{{\left(\mathrm{SAVI}\right)}^{2}+{\left(\mathrm{VanZyl}\_\mathrm{vol}\_g\right)}^{2}}$$

**Figure 11:**
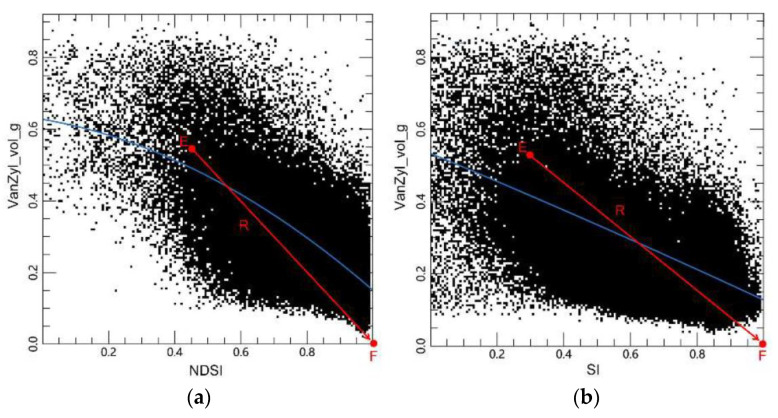
Construction of the salt-radar models: (**a**) NDSI-VanZyl_vol_g feature space; (**b**) SI-VanZyl_vol_g feature space. (The blue line is the trend line between the components; the red line R is the distance from any point E in the feature space to F (1,0)).

Similar to the models based on NDVI-Van_vol_g and SAVI-Van_vol_g feature spaces, models based on the NDSI-Van_vol_g and SI-Van_vol_g feature spaces were constructed. As shown in Figure 11, there was a significant linear relationship between NDSI-Van_vol_g and SI-Van_vol_g. Using the distance from any point in the salt-radar feature space to point F (1, 0) can effectively distinguish different salinity levels of land. The distance R from point E (i.e., a point in the feature space) to point F can be calculated by:

(10) $$R=\sqrt{{\left(\mathrm{NDSI}-1\right)}^{2}+{\left(\mathrm{VanZyl}\_\mathrm{vol}\_g\right)}^{2}}$$

(11) $$R=\sqrt{{\left(\mathrm{SI}-1\right)}^{2}+{\left(\mathrm{VanZyl}\_\mathrm{vol}\_g\right)}^{2}}$$

The ORSDI based on the NDSI-Van_vol_g and SI-Van_vol_g feature space can be, respectively, calculated by:

(12) $${\mathrm{ORSDI}}_{3}=\sqrt{{\left(\mathrm{NDSI}-1\right)}^{2}+{\left(\mathrm{VanZyl}\_\mathrm{vol}\_g\right)}^{2}}$$

(13) $${\mathrm{ORSDI}}_{4}=\sqrt{{\left(\mathrm{SI}-1\right)}^{2}+{\left(\mathrm{VanZyl}\_\mathrm{vol}\_g\right)}^{2}}$$

## 5. Results

### 5.1. Soil Salinity Inversion

**Figure 12:**
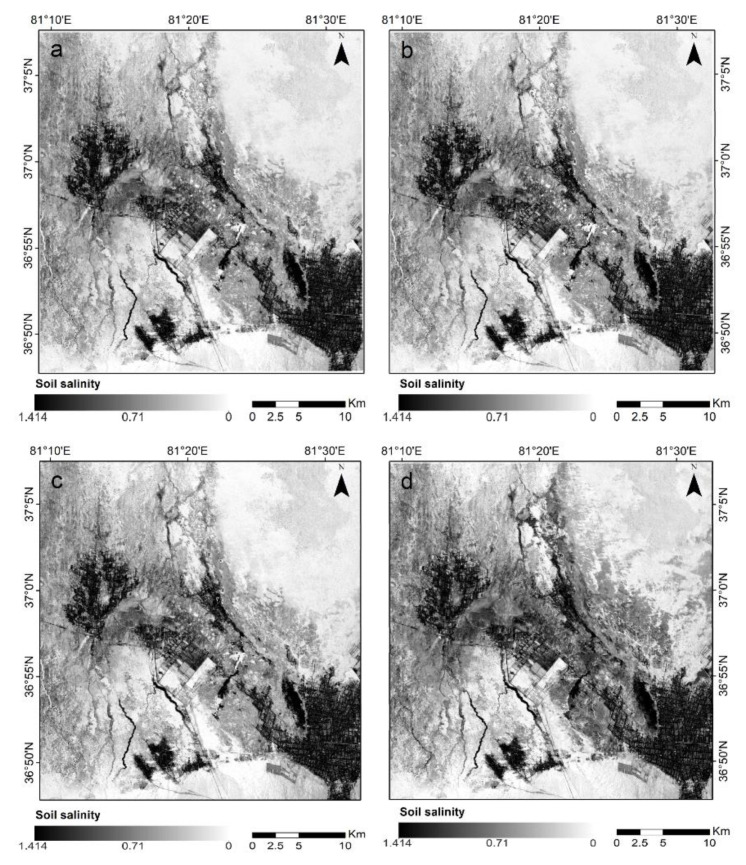
Soil salinity inversion results of the study area: (**a**) ORSDI1; (**b**) ORSDI2; (**c**) ORSDI3; (**d**) ORSDI4.

To explore the distribution of soil salinity throughout the study area, the four constructed soil salinity inversion models (i.e., ORSDI_1_, ORSDI_2_, ORSDI_3,_ and ORSDI_4_) were used, and soil salinity inversion was performed in the entire study area, as shown in Figure 12. The deeper the color on the image, the lower is the salinity degree, while the lighter the color, the higher is the salinity degree.

### 5.2. Accuracy Validation of Soil Salinity Inversion Model

**Figure 13:**
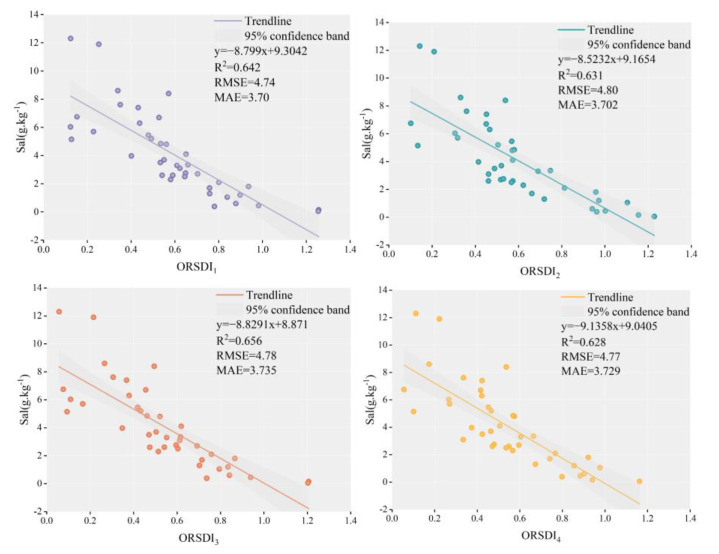
Correlation analysis between the model inversion results and measured data.

To analyze the applicability of four soil salinity inversion models for salinity monitoring in the Keriya Oasis, four soil salinity inversion models were validated and compared using the measured salinity data from 40 measurement points on the field survey, the salinity range of soil samples is 0.05–12.3 (g/kg), 0.17–20.6 (dS/m), of which the number of soil samples in non-saline land is five, the number of soil samples in mildly saline land is 12, the number of soil samples in moderately saline land is nine, and the number of soil samples in severely saline land is 14, for a total of 40 soil samples. The results shown in Figure 13 indicate that the correlation with soil salinity of the four soil salinity inversion models was greater than 0.6. The ORSDI_3_ model achieved the highest correlation, having an R^2^ of 0.656; it was followed by the ORSDI_1,_ ORSDI_4_, and 0RSDI_2_ models, which achieved R^2^ values of 0.642, 0.628, and 0.631, respectively. These results indicate that the proposed method of soil salinity inversion has a relatively efficient information extraction ability and can accurately reflect the distribution of salinity in the study area.

To distinguish different degrees of soil salinity, the natural breaks method of ArcGIS 10.7, which can maximize differences between individual features and can fully consider the histogram distribution of the inversion model [87,88], was used, and the ORSDI was divided into four categories as shown in Table 5: non-saline areas (water and plant cover), mildly saline areas, moderately saline areas, and heavily saline areas.

**Figure 14:**
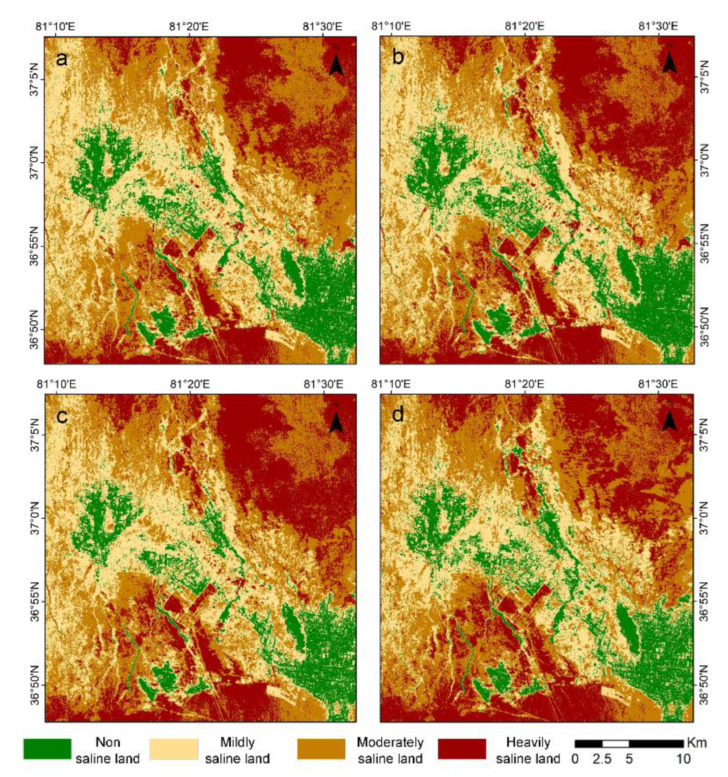
Spatial distribution of soils with different salinity levels based on different feature space models: (**a**) ORSDI1; (**b**) ORSDI 2; (**c**) ORSDI 3; (**d**) ORSDI4.

As shown in Figure 14, soil salinity was widely and discontinuously distributed in the study area. At the same time, different degrees of soil salinity showed large differences in spatial distribution. In the whole study area, the salt content of the surface soil was high in the periphery but low in the middle. The non-salinized areas were mainly located in the western and southeastern parts of the study area and near the banks of the Keriya River. Slightly saline soils were distributed in the transition zone between the non-salinized and moderately saline soils. The region with moderate salinity was the most widely distributed. Severely salinized soils were mainly distributed in the northeastern and southwestern parts of the study area.

## 6. Discussion

### 6.1. Accuracy Analysis of Inversion Models

It can be seen from Figure 13 that the correlation between ORSDI and the measured salinity was larger than 0.6, and the proposed soil salinity inversion model based on the Landsat 8 OLI and PALSAR-2 data could meet the requirements of soil salinity, inversing to a certain extent. The inversion accuracy of the proposed model was approximately 0.6; this could be because the ALOS PALSAR-2 radar image speckle noise was not completely removed after filtering and multi-looking processing. The analysis also showed some salt-tolerant vegetation was present in the heavily saline areas, resulting in the vegetation indices of these areas were not as small as expected, which could be the reason for the lower inversion accuracy of the proposed model.

### 6.2. Spatial Distribution of Soil Salinity

As presented in Figure 14, the overall spatial distribution in surface salinity of the oasis soils showed higher levels of soil salinity in the northeast than in the southeast, with insignificant differences in the southwest and northwest. In addition, there were local anomalies in the study area, most of the saline land was located at the edge of the oasis and in the interlaced part of the desert, and northern part of the study area, which could be because the northern part of the Keriya Oasis received more soluble salts washed out from the upper Keriya River.

In addition, the topography was high in the south but low in the north, which could contribute to the movement of groundwater to the northern part of the region, where the soils had the highest electrical conductivity. Using the proposed model, it is also possible to analyze a mixture of heavily saline, moderately saline, mildly saline, and non-saline soils within the study area with a sporadic distribution of saline soils; most of these sporadic distributions relate to agricultural land within the oasis area or on the natural cover in the oasis center. The non-salinized and mildly salinized areas were mainly located near the Keriya river, which could be due to the good irrigation and drainage facilities near the river and years of land improvement that have reduced the salinity content of the soil.

Groundwater plays a dominant role in the accumulation of soil salts, especially the depth of burial of groundwater, which is directly related to the ability of the capillary soil water to reach the soil surface and cause the accumulation of salts in the soil, thus affecting the degree of soil salinity [89]. The effect of the groundwater on the northern part was significantly greater than that of the central and southern regions, mainly due to the shallower groundwater depth in the north and a larger amount of water rising to the soil through the capillary water under the effect of evaporation, as well as stronger downward leaching of salts.

### 6.3. Uncertainty Analysis

Although the proposed soil salinity inversion model achieved good results in the test, it utilizes only part of the optical remote sensing indexes and retains only part of the polarization feature components of the PALSAR-2 data, thus inevitably losing certain useful polarization information. However, other scattering polarization characteristics should be further explored, and the physical mechanisms of different polarization characteristic components obtained from different polarization decompositions of the PALSAR-2 data and their quantitative relationships with the soil salinity need to be further investigated.

The NDVI has been widely used in the analysis of the salinization processes [90,91]. However, in areas with a low vegetation cover, the NDVI is strongly influenced by the soil background and thus may underestimate some vegetation information. The optical remote sensing index used in the present study, the SAVI, can reduce the effect of soil background [78,92], but cannot eliminate it. This might be one of the reasons why the inversion results presented in this paper are not very high compared to previous studies that used optical data in constructing soil salinity inversion models based on feature space. For instance, Lu Jing et al. [93] constructed a modified salinization detection index (MSDI) using SI and modified type of soil adjusting the vegetation index (MSAVI), and the results showed that the correlation between MSDI and soil salinity was 0. 85; Bing Guo et al. [94] using vegetation indices–salinity indices constructed soil salinity monitoring indicators, the experimental results showed that the remote sensing monitoring index constructed based on the ENDVI-SI_4_ feature space had the correlation with soil salinity (R^2^ = 0.719).

Based on previous studies, the correlation between the feature space soil salinity inversion model and soil salinity was found to be higher when the salinity index was used than when it was not used. For example, the correlation between the remote sensing inversion model of soil salinity constructed by Ding et al. [95] using MSAVI and wet index and soil surface salinity (with R^2^ = 0.84) was lower than that of the inversion model of soil salinity constructed by Bing Guo et al. [96] using MSAVI-SI with R^2^ = 0.89. The proposed model’s validation results have also shown that the correlation of the feature-space model of the salt-radar model may be higher, probably because the SI and NDSI represent a direct reflection of soil salinity, while the vegetation index indicates an indirect reflection of soil salinity. The proposed model’s validation results have also shown that the correlation of the feature-space model of the salt-radar model may be higher, probably because the SI and NDSI represent a direct reflection of soil salinity, while the vegetation index indicates an indirect reflection of soil salinity.

The polarization decomposition establishes several different scattering mechanisms based on the polarization matrix, which could extract different polarization characteristic components with obvious physical significance and targeting [72,97]. The polarization characteristic component could provide the scattering mechanism of the land cover [80]. Volume scattering is dominant in vegetated areas [72], so information on the change in the land surface vegetation in an arid zone can reflect the regional soil salinity status [98].

As mentioned before, soil salinity is influenced by a combination of many factors, including climate, vegetation, topography hydrology, soil moisture, soil roughness, etc., [75,99,100]. However, not all affecting factors of soil salinity were considered in this study, and more influencing factors will be analyzed in future work to investigate the distribution of soil salinity and improve the inversion accuracy.

Although the proposed soil salinity inversion model showed good performance in the typical arid zone of the Keriya region, geographical and ecological conditions could differ among different regions, so mechanisms, manifestations, types, and extent of soil salinity could vary considerably. Therefore, the proposed ORSDI model needs to be further verified for different study areas and scales to prove its feasibility and practicability.

## 7. Conclusions

Soil salinity is a key factor affecting the stability of oases and the quality of the ecological environment in arid zones and has been the greatest challenge hindering the sustainable development of agricultural production in Xinjiang’s oases [95]. Applying the principles and methods of remote sensing, this study aims to extract information on regional soil salinity.

The main contributions of this study can be summarized as follows:

(1) This study develops a soil salinity inversion method using optical surface indexes and radar polarization feature components to form a feature space. Based on the special surface cover and natural environment of the Keriya Oasis, four typical optical surface indexes—the NDVI, SAVI, NDSI, and SI—are quantitatively inverted using the Landsat 8 OLI remote sensing data, the ALOS PALSAR-2 radar remote sensing data are decomposed according to seven types of polarization, and an optimal polarization decomposition component is extracted.
(2) In this study, different multi-source remote sensing data are deeply exploited, and different feature parameters in the soil salinization process are considered. In addition, four two-dimensional optical-radar feature spaces—NDVI-VanZyl_vol_g, SAVI-VanZyl_vol_g, NDSI-VanZyl_vol_g, and SI-VanZyl_vol_g—are constructed using the optical feature parameters (i.e., NDVI, SAVI, NDSI, and SI) and the optimal radar feature component (VanZyl_vol_g).
(3) A soil salinity remote sensing-based inversion model, the ORSDI, is constructed by analyzing the distribution regularity of soils with different degrees of salinity in the feature space. The accuracy of the models was validated against 40 measured salinity datasets. The results show that the trend of the ORSDI values is consistent with that of the field measured data. ORSDI_1_ achieves an R^2^ of 0.642, ORSDI_2_ has an R^2^ of 0.631, ORSDI_3_ achieves an R^2^ of 0.656, and ORSDI_4_ has an R^2^ of 0.628. This proposed model can provide rapid and relatively accurate monitoring results of oasis soil salinity. Therefore, it has a certain potential for the extraction and dynamic monitoring of saline land information in arid areas.

## Figures and Tables

**Table 1:** Classification system of salinization.

Salinization Degree	EC (dSm^−1^)	Salinity (g·kg^−1^)	Surface Vegetation Type
Non-saline soil	0–2.0	<1.0	Arable land with good crops, trees, shrubs, grassland and reed land.
Mildly saline soil	2.0–4.0	1.0–3.0	Spread between non-salted land, vegetation coverage of approximately 15–30%.
Moderately saline soil	4.0–8.0	3.0–5.0	Patchy distribution; vegetation coverage around 10–15%.
Heavily saline soil	8.0–16.0	5.0–10.0	Obvious salt crusts; only salt-tolerant plants, vegetation cover around 5–10%.

Note: The classification was performed according to the “Work Outline about Reuse of Salted Soil at the County Level in Xinjiang”.

**Table 2:** Polarimetric features obtained from the PALSAR-2 data.

Decomposition Features	Symbol	Number of Parameters	Polarimetric Parameter
Pauli	Pauli	3	Pauli_surf_b, Pauli_vol_g, Pauli_dbl_r
Freeman	Free	3	Freeman_surf_b, Freeman_vol_g, Freeman_dbl_r
Freeman Durden	FD	3	Freeman Durden_surf_b, Freeman Durden_vol_g, Freeman Durden_dbl_r
Cloude	Cloude	3	Cloude_surf_b, Cloude_vol_g, Cloude_dbl_r
Sinclair	Sin	3	Sinclair_surf_b, Sinclair_vol_g, Sinclair_dbl_r
VanZyl	Van	3	VanZyl_surf_b, VanZyl_vol_g, VanZyl_dbl_r
Yamaguchi	Yam	4	Yamaguchi_surf_b, Yamaguchi_vol_g, Yamaguchi_dbl_r, Yamaguchi_hlx

**Table 3:** Vegetation and soil salinity indexes for soil salinity assessments.

Category	Index	Formulation	Reference
Vegetation index	NDVI	(NIR − R)/(NIR + R)	[79]
	SAVI	[(NIR − R)/(NIR + R + L)] × (1 + L)	[78]
Salinity index	SI	$\sqrt{B\times R}$	[28]
	NDSI	(R − NIR)/(R + NIR)	[28]

B: Blue band, R: Red band, NIR: Near-infrared band. L is a soil adjustment factor; T_max_ = a + b × NDVI; T_min_ = c + d × NDVI, where a, b, c, d are fitting coefficients of dry and wet edges.

**Table 4:** The SNR values of the feature components (dB).

	Cloude	Freeman	Freeman Durden	Pauli	Sinclair	VanZyl	Yamaguchi
Surf_b	356.067	308.391	252.007	225.301	203.320	240.865	268.901
Vol_g	28.8860	412.218	378.320	213.868	225.270	373.967	268.092
Dbl_r	72.6462	248.521	332.305	221.481	217.883	260.327	329.654
Hlx							140.923

**Table 5:** Thresholds of different levels of soil salinity.

Salinization Grade	ORSDI1	ORSDI2	ORSDI3	ORSDI4
Heavy	<0.15	<0.17	<0.14	<0.21
Moderate	0.15~0.46	0.17~0.45	0.14~0.45	0.21~0.70
Mild	0.46~0.91	0.45~0.88	0.45~0.92	0.70~0.98
Non	0.91~1.41	0.88~1.41	0.92~1.41	0.98~1.41

## Data Availability

Not applicable.
